# Home Based Exercise Rehabilitation Programs to Prevent Physical Frailty and Hospitalization-Associated Disability

**DOI:** 10.14789/jmj.JMJ23-0034-P

**Published:** 2023-12-22

**Authors:** TETSUYA TAKAHASHI, TOMOYUKI MORISAWA, MASAKAZU SAITOH, KOTARO IWATSU, TOSHIYUKI FUJIWARA, HIROYUKI DAIDA

**Affiliations:** 1 Department of Physical Therapy, Faculty of Health Science. Juntendo University, Tokyo, Japan; 1 Department of Physical Therapy, Faculty of Health Science. Juntendo University, Tokyo, Japan; 2 Department of Physical Therapy, Juntendo University Graduate School of Health Science, Tokyo, Japan; 2 Department of Physical Therapy, Juntendo University Graduate School of Health Science, Tokyo, Japan; 3 Department of Rehabilitation Medicine, Juntendo University Graduate School of Medicine, Tokyo, Japan; 3 Department of Rehabilitation Medicine, Juntendo University Graduate School of Medicine, Tokyo, Japan; 4 Juntendo University Graduate School of Health Science, Tokyo, Japan; 4 Juntendo University Graduate School of Health Science, Tokyo, Japan

**Keywords:** home exercise, prevention, physical frailty, hospitalization-associated disability

## Abstract

Daily health management and exercise are important for staying healthy and avoiding the need for long-term care. However, it is not easy to maintain regular exercise. Therefore, exercise needs to be done efficiently. In recent years, due to the aging population and increasing severity of illness, older patients often experience a significant decline in physical function, even with minimal rest, which often interferes with their daily life after discharge from the hospital. Frailty not only affects ADLs, but also strongly influences prognosis, including the development of atherosclerotic disease and rehospitalization. This perspective is a summary of the 51st Metropolitan Public Lecture held on June 17, 2023, and discusses exercise-based rehabilitation programs that can be delivered at home to prevent physical frailty and avoid hospitalization-related disability.

## Introduction

Without going back to Marcus Tullius Cicero's (BC106~BC43) “Essay on Old Age”, it is obvious that moderate exercise is essential for the prevention of age-related muscle wasting and weakness. In 1990, when the author had just become a physical therapist, the New England Journal of Medicine published a paper entitled “Exercise training and nutritional supplementation for physical frailty in very elderly people^1)^”, which was deeply etched in my inexperienced mind. The content of the study was that resistance training (80% of maximum repetitions per session) targeting muscles important for activities of daily living (ADL) was performed three times a week for 10 weeks on older residents of a nursing home with an average age of 87.1 years, resulting in an increase in muscle strength of 113 ± 8% and an increase in walking speed of 11.8 ± 3.8%. I remember being surprised to learn that muscle mass could be increased by resistance training even in the older population. In addition, it has been reported that resistance training causes a shift from type IIb fibers to type IIa fibers in the older population, along with an increase in muscle strength and muscle mass^2)^, and the fact that the muscle adaptation of the older populations by resistance training is similar to that of younger people has even become a cornerstone of the author's thinking when conducting physical therapy.

Since Rosenberg^3)^ defined sarcopenia in 1989, frailty and sarcopenia associated with loss (wasting) and reduction of skeletal muscle have been of great interest in rehabilitation medicine, coupled with an aging patient population. In addition to sarcopenia, “dynapenia”, derived from the Greek words dyna (strength) and penia (decrease), has recently gained attention^4)^. Sarcopenia is diagnosed by age-related muscle loss, muscle weakness, and decreased walking speed, but muscle strength is not originally a function dependent solely on muscle mass (muscle wasting). In addition to muscle cross-sectional area (muscle mass), muscle strength is affected by an increase in the type and total number of motor units mobilized (recruitment), an increase in the frequency of alpha motor nerve firing (rate coding), and the involvement of the nervous system in the regulation of motor unit activity phases (synchronization). Dynapenia is described as “the age-related loss of muscle strength and power”, but since muscle strength and muscle power (strength x speed) are different to begin with, further research is expected in the future.

Originally, in the field of rehabilitation medicine, in addition to comprehensive assessment of physical function, it is necessary to explore possible life-related problems through comprehensive geriatric assessment by a multidisciplinary team for life functions and activities, and develop a personalized rehabilitation program that integrates the environment.

## Preventing frailty at home

Frailty is a comprehensive health concept that includes not only the decline in physical and mental function associated with aging, but also cognitive decline, depression, loneliness, and poor nutrition. Among them, physical frailty is the most common and has a significant impact on healthy life expectancy^5)^.

To understand “physical frailty”, Fried's diagram of the frailty cycle is very helpful^5)^. First, “loss of independence” means “need for long-term care”. To avoid the state of needing long-term care, we must prevent the upstream disability. Disability is related to abilities such as “walking speed (ability to walk fast)”, “muscle strength and power”, and “VO2max (endurance)”. To maintain this “ability to walk fast,” “muscle strength,” and “endurance,” training that focuses on ankle and hip flexibility (stretching), especially leg strength (muscle training), and endurance (aerobic exercise) is necessary.

In addition, changes in physical condition due to aging, various diseases, and loss of appetite can cause loss of muscle mass, or “weight loss”. One of the causes of this loss of muscle mass is “decreased physical activity”. Furthermore, “decreased physical activity” is affected by “decreased walking speed” and as mentioned above, “decreased walking speed” is affected by muscle strength and endurance. So these are interrelated in a cycle, and the vicious cycle needs to be broken somewhere. To achieve this, 1) muscle training and 2) aerobic exercise are necessary.

## Muscle training to increase muscle strength

Muscle strength is essential for human daily physical activities. By increasing muscle strength, people can perform a single movement with less effort. So-called “muscle training” is important for increasing muscle strength. Professionally, it is sometimes referred to as “resistance training”^6)^.

Resistance training uses resistance and load on the muscles through the use of weight machines, rubber tubing, or own body weight to increase muscle size, activate nerves that facilitate muscle strength, and improve overall muscle function.

To perform muscle training safely and effectively, it is important to follow the rules^6)^.

### 1) Muscle training precautions

The following precautions should also be taken^6)^.

• Perform preparatory exercises and avoid overexertion from the start.

• Exercise large muscles first.

• Do not fully extend elbows and knees.

• Exercise with proper form and be aware of the muscles.

• Hold the grip lightly as it may cause an excessive increase in blood pressure.

• Do not hold breath.

• Exhale when lifting the weight and inhale lowering the weight.

• Lift and lower the weight slowly for about 4 seconds.

• Rest between exercises.

• Stop exercising immediately if experience dizziness, irregular pulse, unusual shortness of breath, or angina-like discomfort.

### 2) Intensity and amount of muscle training (Number of repetitions x sets x frequency)^6)^

To strengthen muscles, it is necessary to place a load on the muscles that is greater than the muscle strength used in everyday life. To set the specific load, find the weight that can be lifted only once (called 1 repetition maximum: 1RM) and calculate and set the load to 30-40% for the upper body and 50-60% for the lower body.

In practice, however, it is often difficult to measure the exact 1RM, so a method is used in which an approximate load is determined and the load is gradually increased while checking to see if 10 repetitions can be performed. This is called the titration method.

It is best to use a load that feels “a little hard” after 10 repetitions. On the other hand, if it doesn't feel “a little hard,” it won't be very effective.

Repeat the same type of exercise for two or three sets. If you start to feel lighter, you can gradually increase the weight. It is said that 2-3 days a week is quite effective.

### 3) Specific methods

**Figure 1:**
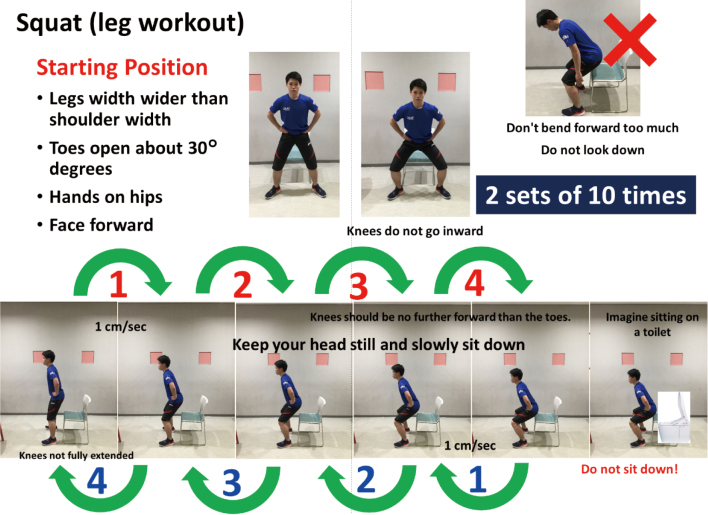
How to perform appropriate squatting exercise

This section describes the most important lower body exercise (squatting) that can be done at home. Do it without jumping and without stopping to breathe. As lowering the hips, it is best to consciously exhale (Figure 1).

## Aerobic exercise to improve endurance^7)^

Endurance is “the ability to move the body for an extended period of time. An energy source is needed to move muscles. Oxygen and nutrients are needed to produce this energy in the body. The more oxygen the body can take in, the more energy it can sustainably produce for muscle contraction. In other words, it is important for the body to be able to take in a lot of oxygen in order to move for a long period of time. Therefore, to use a more technical term, “endurance” can be defined as “the ability of the body to take in oxygen through exercise”.

Aerobic exercise is exercise that can be performed continuously for long periods of time, such as walking or cycling. Because individual has a different level of fitness, it is important to determine the frequency, intensity, duration, and type of exercise according to the individual's fitness level. People with a pre-existing heart condition or those who have been hospitalized for a medical condition should consult their physician before exercising.

**Figure 2:**
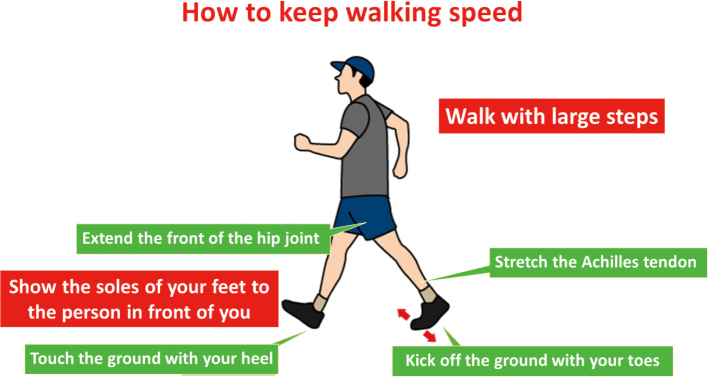
How to keep walking speed

When walking as an aerobic exercise to improve endurance, take a slightly larger stride and walk to maintain an effective walking posture and walking speed (Figure 2).

Frequency (F), intensity (I), duration (T) and type of exercise (T) are referred to by the acronym “FITT”.

## Four key points of aerobic exercise^7)^

• Frequency: 3 to 5 times a week. If possible, do it every day.

• Intensity: The intensity should be easy to moderately strenuous, and should be able to exercise while talking.

• Time: 20 to 30 minutes. If it is difficult to exercise continuously, it is said that the effect is the same if the exercise is divided into several sessions so that the amount of exercise per day is 20 to 30 minutes.

• Type: Walking, cycling, aerobic exercise, etc. are suitable.

## Muscle training for older patients with frailty

In Japan, there has been a trend toward shortening the length of stay in acute care hospitals. In recent years, due to the aging of the target patient population and the increasing severity of illness, frail and older patients often experience a marked decline in physical function even with short time bed rest, which often interferes with their daily lives after discharge from the hospital. Frailty and physical functional decline not only affects ADLs, but also strongly influences prognosis, including the development of atherosclerotic disease and rehospitalization.

More recently, due to the aging of patients, there is a significant number of patients whose exercise function cannot be fully restored within a short hospital stay and who are unable to return to their pre-hospital exercise function^8)^. These patients have become known as “Hospitalization-Associated Disability” or “Hospitalization-Acquired Disability” (HAD)^9)^. To ensure a more efficient recovery with a shorter hospital stay, optimal exercise programs need to be further investigated.

The usual rehabilitation program is a step-by-step program that gradually increases gravity loads and movement, such as lifting, sitting, standing, and walking, after absolute rest is lifted, but in older patients with frailty, in addition to increasing walking distance, a rehabilitation program to improve ADL functions, such as the ability to stand up safely and stand stably with good balance. Knee extension exercises are often performed with the patient in a bed-sitting position to strengthen lower extremity muscles. However, it is easier to achieve the specificity of the desired training effect by repeating ADL activities such as standing and sitting than by simply repeating knee extension.

The Japanese Geriatrics Society defined frailty in 2014 as follows^10)^: Frailty is a state of increased vulnerability to stress due to a decline in physiological reserve capacity in old age, which may lead to lifestyle dysfunction, need for care, and death. The concept includes not only physical problems such as loss of mobility due to muscle weakness and increased susceptibility to falls, but also mental and psychological problems such as cognitive dysfunction and depression, and social problems such as living alone and economic deprivation.

Focusing on physical frailty, the following three points are important for an exercise program for older patients with frailty.

• Overcoming muscle weakness

• Preventing loss of mobility

• Preventing falls

Currently, the Short Physical Performance Battery (SPPB)^11)^ is widely used worldwide in the assessment of physical frailty, and each domain of the SPPB has three components: standing balance, walking speed, and leg strength (power). These three components of the SPPB are precisely what characterize the assessment of physical frailty. Therefore, at Juntendo University Hospital, these three SPPB components plus the walking distance (endurance) component are evaluated regularly on a daily basis to standardize the training menu (Table 1)^12)^. For example, if the patient can perform semi-tandem standing for 10 seconds in the balance test, but tandem standing is difficult, the 4-meter walking time is slow at 9.2 seconds, and the patient cannot stand without upper limb support (can stand using a handrail) in the standing test. The patient can walk 30 meters down a corridor in the ward. In such a case, the performance level of muscle strength, balance, movement and endurance is as shown in Table 1, and it will be easy to see where the patient's physical function is most impaired.

**Table 1:** Short Physical Performance Battery (SPPB) and Juntendo original scale

SPPB	BASE	Balance (10-s balance tests)	Ambulation (a timed 4-m walk)	Sit-ups (a timed 5 times-repeated chair sit-to-stand test)	Endurance (maximum walking distance)
4	5	One leg 10s	Less than 4s	< 9.2 s	340 m (6 min)
	4	Full-tandem 10s	Less than 4.82s	< 11.19 s	180 m
3	3	Full-tandem 3 ~ 9.99s	4.82 ≤ 6.20s	11.2 ≤ 13.69 s	80-179 m
2	2	Semi-tandem 10s	6.21 ≤ 8.70s	13.7 ≤ 16.69 s	40 m
1	1	Side-by-side, 10s	More than 8.70s	> 16.70 s	15 m
0	0.8	Side-by-side, 3 ~ 9.99s	4m with light assistance	> 60 s or stand up 2-5 times	4 m
	0.6	Broad base 10s (without support)	4m with moderate assistance	Stand up once without support	Wheelchair 30 min
	0.4	Able to stand with support	4m with heavy assistance	Stand up with arm support	Wheelchair 10 min
	0.2	Able to stand with assistance	2.3 steps with heavy assistance	Stand up with assistance	Able to sit on the edge of bed
	0	Unable to stand	Unable to walk	Unable to stand up	Bed rest

SPPB 0-6: low function, 7-9: moderate function, 10-12: high function.

**Figure 3:**
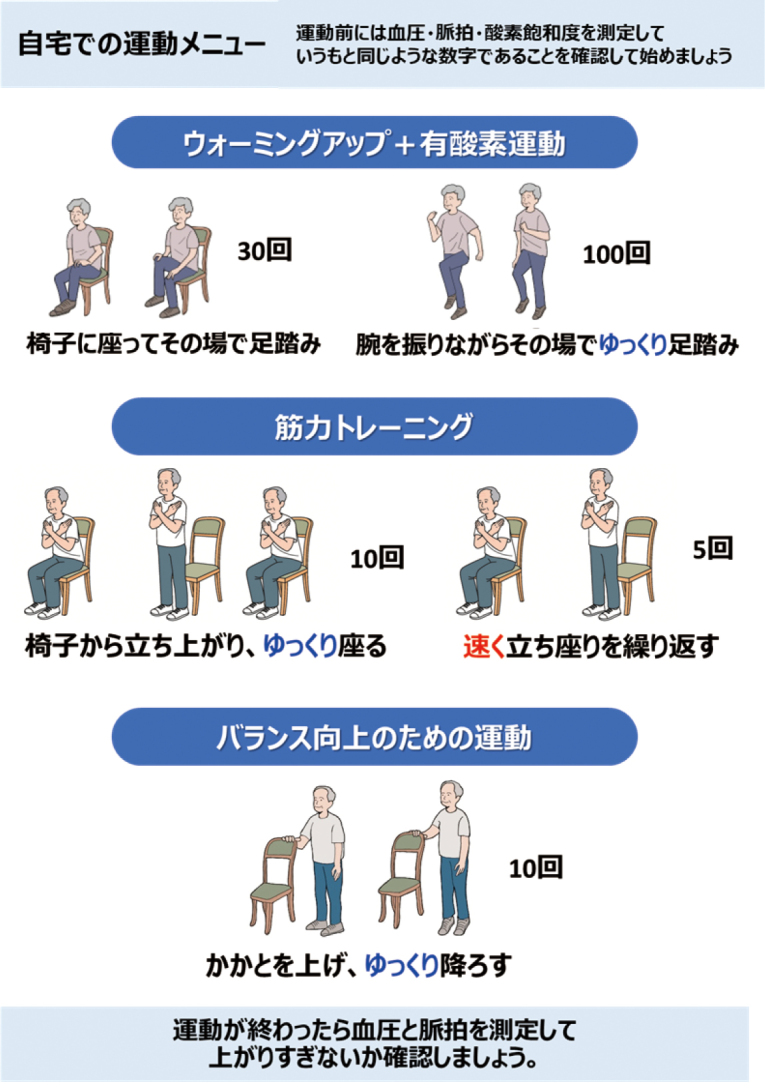
Home exercise program for frailty prevention

By visually presenting the results of such an assessment, patients themselves can easily understand the decline in their abilities, leading to adherence to exercise therapy. With the current trend to reduce the length of stay in acute care hospitals, this system will be used as a standard HAD prevention training program to be implemented immediately after hospitalization with a view to discharge. Then, at the time of discharge, physical therapists provide discharge guidance with awareness of these four components to prevent progression of frailty (Figure 3).

The five-chair rise test is important for overcoming the dynapenia described at the beginning of this article and is one of the exercises that should be consciously performed in addition to regular muscle training, especially in patients with frailty.

## Tele-cardiac rehabilitation

Currently, access to medical facilities for the older people is a problem due to geographical and time constraints, economic issues, lack of caregivers and carers, and uneven distribution of medical resources. In addition, many people are reluctant to go out due to recent new coronavirus infections, and the problem of difficulties in visiting hospitals is a major issue for all generations^13)^. These difficulties are not limited to general outpatient visits, but also extend to rehabilitation for the recovery of functional disabilities caused by various diseases, traumas, and pathological conditions^14)^.

In Japan, “tele-cardiac rehabilitation” conjures up images of so-called “real-time online exercise” using the Internet, but in reality, “tele-cardiac rehabilitation” devices and systems are used in a variety of ways, and there is no clear definition. In general, “telemedicine” is based on an infrastructure using computer and communication technology, but by distinguishing between “asynchronous (store-and-forward, asynchronous)” and “synchronous (real-time, synchronous)” telerehabilitation^15)^.

In Japan, the synchronous model is interpreted as a model that provides comprehensive exercise program, patient education, and psychological support similar to those provided in outpatient program, while monitoring real-time biometric data such as electrocardiogram and blood pressure, in addition to video communication over the Internet^16, 17)^. Tele-cardiac rehabilitation does not require an exercise bike for exercise. Many older patients require more basic exercise than cycling on an exercise bike. Especially in the immediate post-discharge period after cardiac surgery, patients are often forced to rest longer than necessary or leave the hospital without a better understanding of their disease or current condition during their short hospital stay. For such patients, we use home-hospital videoconferencing to monitor their physical condition, subjective symptoms, daily blood pressure, steps, weight change, facial expression, lower extremity edema, and median incision site while monitoring their ECG, blood pressure, and oxygen saturation in real time. In addition to thorough pre-orientation, limiting the tablet's functions, minimizing the use of apps, and keeping the procedure simple, even elderly patients are able to complete the procedure without major operational problems.

## Conclusion

This perspective paper has described the importance of preventing physical frailty and specific approaches, particularly exercise-based rehabilitation programs, that can be implemented to minimize hospitalization-related disability in frail older patients, as well as the current state of telerehabilitation.

## Funding

This work was supported by JSPS KAKENHI Grant Number JP20H04055.

## Author contributions

TT gave a lecture at the 51st Metropolitan Public Lecture, which was summarized in the manuscript. MT, SM, and IK collected relevant information and provided relevant content advice on the manuscript; FT and DH provided clinical and research advice on the manuscript. All authors read and approved the final version of the manuscript.

## Conflicts of interest statement

The authors declare that they have no competing interests related to this manuscript.
